# Genome-Wide Discovery of Genes Required for Capsule Production by Uropathogenic *Escherichia coli*

**DOI:** 10.1128/mBio.01558-17

**Published:** 2017-10-24

**Authors:** Kelvin G. K. Goh, Minh-Duy Phan, Brian M. Forde, Teik Min Chong, Wai-Fong Yin, Kok-Gan Chan, Glen C. Ulett, Matthew J. Sweet, Scott A. Beatson, Mark A. Schembri

**Affiliations:** a School of Chemistry and Molecular Biosciences, University of Queensland, Brisbane, Queensland, Australia; b Australian Infectious Diseases Research Centre, University of Queensland, Brisbane, Queensland, Australia; c Australian Centre for Ecogenomics, University of Queensland, Brisbane, Queensland, Australia; d Division of Genetics and Molecular Biology, Institute of Biological Sciences, Faculty of Science, University of Malaya, Kuala Lumpur, Malaysia; e School of Medical Science and Menzies Health Institute Queensland, Griffith University, Gold Coast, Australia; f Institute for Molecular Bioscience, University of Queensland, Brisbane, Queensland, Australia; Washington University School of Medicine

**Keywords:** bacteriophages, capsule, molecular genetics, regulation of gene expression, sepsis, urinary tract infection, uropathogenic, *E. coli*, virulence

## Abstract

Uropathogenic *Escherichia coli* (UPEC) is a major cause of urinary tract and bloodstream infections and possesses an array of virulence factors for colonization, survival, and persistence. One such factor is the polysaccharide K capsule. Among the different K capsule types, the K1 serotype is strongly associated with UPEC infection. In this study, we completely sequenced the K1 UPEC urosepsis strain PA45B and employed a novel combination of a lytic K1 capsule-specific phage, saturated Tn*5* transposon mutagenesis, and high-throughput transposon-directed insertion site sequencing (TraDIS) to identify the complement of genes required for capsule production. Our analysis identified known genes involved in capsule biosynthesis, as well as two additional regulatory genes (*mprA* and *lrhA*) that we characterized at the molecular level. Mutation of *mprA* resulted in protection against K1 phage-mediated killing, a phenotype restored by complementation. We also identified a significantly increased unidirectional Tn*5* insertion frequency upstream of the *lrhA* gene and showed that strong expression of LrhA induced by a constitutive Pcl promoter led to loss of capsule production. Further analysis revealed loss of MprA or overexpression of LrhA affected the transcription of capsule biosynthesis genes in PA45B and increased sensitivity to killing in whole blood. Similar phenotypes were also observed in UPEC strains UTI89 (K1) and CFT073 (K2), demonstrating that the effects were neither strain nor capsule type specific. Overall, this study defined the genome of a UPEC urosepsis isolate and identified and characterized two new regulatory factors that affect UPEC capsule production.

## IMPORTANCE

Urinary tract infections (UTIs) are among the most common bacterial infections in humans and are primarily caused by uropathogenic *Escherichia coli* (UPEC). Many UPEC strains express a polysaccharide K capsule that provides protection against host innate immune factors and contributes to survival and persistence during infection. The K1 serotype is one example of a polysaccharide capsule type and is strongly associated with UPEC strains that cause UTIs, bloodstream infections, and meningitis. The number of UTIs caused by antibiotic-resistant UPEC is steadily increasing, highlighting the need to better understand factors (e.g., the capsule) that contribute to UPEC pathogenesis. This study describes the original and novel application of lytic capsule-specific phage killing, saturated Tn*5* transposon mutagenesis, and high-throughput transposon-directed insertion site sequencing to define the entire complement of genes required for capsule production in UPEC. Our comprehensive approach uncovered new genes involved in the regulation of this key virulence determinant.

## INTRODUCTION

Urinary tract infections (UTIs) are among the most common human bacterial infections and cause significant morbidity, with roughly 175 million cases estimated to occur annually across the globe (1). It is estimated that up to 50% of women will develop a UTI in their lifetime, and UTIs account for >1 million hospitalizations and ~$3.5 billion in medical and societal expenditure each year in the United States alone (2, 3). UTIs usually involve infection of the bladder (cystitis) but can also develop into kidney infection (pyelonephritis) and bloodstream infection (urosepsis). Recurrent UTIs are also of major concern; ~20 to 30% of women with an acute UTI experience a relapsing episode within 3 to 4 months (3), and these infections are frequently associated with antibiotic resistance.

Approximately 75% of all UTIs are caused by uropathogenic *Escherichia coli* (UPEC) (2). UPEC strains largely belong to the *E. coli* phylogenetic group B2 or D and are often clonal, with the most common sequence types (STs) isolated worldwide being ST69, ST73, ST95, and ST131 (4). UPEC possesses a range of virulence factors, including fimbrial adhesins, secreted toxins, iron acquisition systems, flagella, and cell surface polysaccharides, that enable them to colonize the urinary tract and cause disease (5, 6). The accessory gene repertoire of UPEC can vary extensively between different strains and clones, leading to the observation that virulence is dependent on a combination of multiple factors. For instance, changes in the regulation of specific virulence factors, such as type 1 fimbriae, have been observed in the globally disseminated multidrug-resistant ST131 clonal group of UPEC (7). The increasing global incidence of UTIs caused by antibiotic-resistant UPEC highlights the need to better understand UPEC pathogenesis (8, 9).

The O antigen and capsule comprise two cell surface polysaccharides that play important roles in UPEC virulence. There are more than 180 different serotypes of the bacterial lipopolysaccharide (LPS), which consists of a conserved lipid A core region and a variable O antigen region (10, 11). Variation in the O antigen is caused by altered sugar residues and linkage patterns within the component repeating subunits. LPS mediates UPEC resistance to human serum, and common O antigen types frequently identified among human UPEC isolates include O1, O2, O4, O6, O7, O8, O16, O18, O25, and O75 (12).

The *E. coli* polysaccharide capsule is also highly variable, with more than 80 different capsule types described (13). *E. coli* capsules are classified into four major groups based on the genetic organization of the capsule gene cluster, as well as their mechanism of biosynthesis and assembly (13, 14). Group 2 capsules composed of different K antigens (e.g., K1, K2, K5, and K100) are commonly expressed by many UPEC strains (15). The genes involved in the biosynthesis of group 2-type capsules are arranged in three distinct regions (13). Regions I (*kpsFEDUCS*) and III (*kpsMT*) are conserved in all group 2 capsule gene clusters and encode a transmembrane complex involved in the export and assembly of the capsular polysaccharides (14, 16, 17). Region II is serotype specific and encodes enzymes responsible for synthesizing the capsular polysaccharide (18). The group 2 gene cluster is transcribed as two polycistronic operons and is driven by two temperature-regulated promoters upstream of regions I and III (19, 20). Transcription of region III initiates upstream of *kpsM* and proceeds through to region II, aided by the antiterminator protein RfaH (20). Several other global regulators are also involved in controlling transcription of the capsule genes, including the histone-like nucleoid-structuring (H-NS) protein, the DNA-binding regulator protein SlyA, the ribosome-binding protein TypA, and integration host factor (IHF) (19, 21, 22).

The capsule provides protection against phagocytosis and complement-mediated killing (23, 24), and its contribution to UPEC virulence is well established. In a mutagenesis screen of UPEC strain CFT073, defects in capsular polysaccharide transport were identified that attenuated pathogenicity in a murine model of UTI (25). Both the K1 and K2 capsules provide protection from complement-mediated killing, which has been demonstrated by increased survival of UPEC compared to isogenic capsule mutants following incubation in human serum and human blood (26, 27). In mice, the K1 capsule is also required for the development of intracellular bacterial communities (IBCs), which are biofilm-like bacterial aggregates that form in superficial bladder epithelial cells during the early stages of acute UTI and contribute to host immune evasion (28). Additionally, the genes involved in capsule synthesis are upregulated by UPEC during UTI (29, 30).

Among the different K capsule types, the K1 serotype is strongly associated with strains that cause UTI, bloodstream infection, and meningitis (31, 32). The K1 capsule is made up of a chain of sialic acid residues that are synthesized by enzymes encoded by genes in region II of the capsule locus (*neuDBACES*). This polysaccharide is identical to the polysialic acid present on some human cells, and hence the K1 antigen is poorly immunogenic due to molecular mimicry (33). Lytic K1 phages have been used as a diagnostic tool to detect the surface expression of the K1 capsule in UPEC; wild-type strains expressing the K1 capsule are rapidly killed upon encountering the phage due to its specific attachment to the capsule, which then triggers a lytic life cycle, whereas unencapsulated mutants are protected against phage-mediated killing (26).

Given the importance of the K1 capsule in virulence, we sought to define the entire complement of genes involved in its biosynthesis by using a novel approach that combined phage-specific killing with a high-throughput unbiased forward genetic screen. To this end, we first assessed the distribution of group 2 capsules among publicly available *E. coli* complete genomes, and we also determined the full genome sequence of the K1 UPEC strain PA45B, which was originally isolated from the blood of a patient with pyelonephritis. Next, a saturated transposon mutant library of PA45B was generated and incubated with a lytic K1 capsule-specific phage, and genes required for capsule biosynthesis were identified *en masse* by using transposon-directed insertion site sequencing (TraDIS). In addition to known capsule genes, our analysis identified two regulators (*mprA* and *lrhA*) which were further characterized by the generation of defined mutants and examination of their phenotypic properties. Overall, our screen defined the complement of genes required for UPEC K1 capsule expression.

## RESULTS

### Distribution of group 2 capsule types in completely sequenced *E. coli* genomes.

To assess the distribution of group 2 capsules among different *E. coli* isolates and to determine whether there is any correlation between capsule type and phylogroup/sequence type, we performed an *in silico* analysis on a collection of 126 completely sequenced genomes publicly available in the NCBI database (see Data Set S1A in the supplemental material). Based on this analysis, 36 strains in the collection possessed a complete group 2 capsule gene cluster, of which K1 represented the most common K type (12/36 strains) (Data Set S1B). Eight strains possessed a group 2 capsule that could not be typed *in silico*, while an additional nine strains contained an incomplete group 2 gene cluster (one to four genes missing).

10.1128/mBio.01558-17.6 DATA SET S1  (a) List of completely sequenced *E. coli* genomes publicly available in the NCBI database and used in this study. (B) Multilocus sequence type, phylogroup, and K type distribution among the 36 completely sequenced *E. coli* genomes possessing a group 2 capsule. (C) List of virulence genes found in PA45B. (D) Summary of methylome analysis for the PA45B chromosome. (E) Genes identified by TraDIS that did not affect capsule production in PA45B. (F) Insertions in intergenic regions identified by TraDIS that influenced capsule production in PA45B. (G) Bacterial strains and plasmids used in this study. (H) Primers used in this study. Download DATA SET S1, XLSX file, 0.1 MB. Copyright © 2017 Goh et al. 2017 Goh et al. This content is distributed under the terms of the Creative Commons Attribution 4.0 International license.

### Genomic analysis of UPEC strain PA45B.

PA45B is part of a previously characterized collection of UPEC strains isolated from the blood of patients presenting with urosepsis at the Princess Alexandra Hospital (Brisbane, Australia) (34). PA45B belongs to ST95 and phylogroup B2. It expresses the K1 capsule, which allowed us to utilize a lytic K1 capsule-specific phage to kill encapsulated mutants in our TraDIS experiment. Moreover, PA45B can be genetically manipulated, which allowed us to generate targeted deletion mutants to validate hits obtained from our TraDIS analysis. The complete genome of PA45B was determined and shown to consist of a single circular chromosome comprised of 5,074,754 bp (50.5% GC content) that encodes 4,745 putative protein-coding genes (GenBank accession number CP021288). Based on *in silico* typing, PA45B is an O2:K1:H7 strain. PA45B possesses an array of UPEC virulence factors, which include several iron acquisition systems, fimbrial adhesins, autotransporter proteins, and the capsule (Data Set S1C). PA45B carries an IncF-type plasmid that we refer to as pPA45B (147,172 bp; 51.6% GC content). Plasmid pPA45B contains 177 predicted protein-coding genes and three genes associated with antibiotic resistance (*aadA1*, *bla*_TEM-1B_, and *sul1*). Methylome analysis of PA45B identified two distinct DNA recognition motifs (Data Set S1D).

### Phenotypic confirmation of PA45B capsule type.

**FIG 1:**
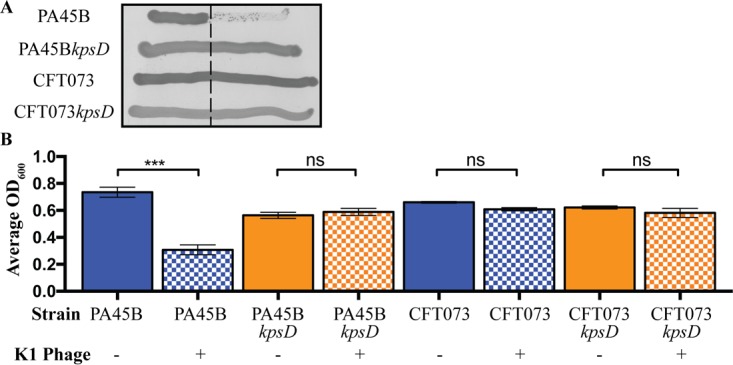
Phenotypic characterization of UPEC strains PA45B (K1 capsule), CFT073 (K2 capsule), and the corresponding capsule mutants (*kpsD*). (A) Cross-brush agar test results, showing K1 phage reaction with PA45B, CFT073, and *kpsD* derivatives. The dashed line indicates the line of phage suspension. (B) Measurement of susceptibility to K1 capsule-dependent phage lysis following growth in microtiter plates. K1 phage was added to cultures grown to an OD_600_ of 0.2 and further incubated for 3 h. The bars represent the OD_600_ at 3 h postinfection. ***, *P* = 0.0001. Data were obtained from three independent experiments; error bars indicate standard deviations. Statistical analysis was performed using an unpaired, two-tailed *t* test.

A lytic K1 phage was used to confirm the expression of the K1 capsule on the surface of wild-type PA45B. Using the standard cross-brush method applied for serotyping, PA45B growth was prevented after contact with the phage suspension (Fig. 1A). The specificity of the phage to the K1 capsule of PA45B was demonstrated through the construction of an isogenic *kpsD* mutant strain which was not susceptible to killing by the K1 phage. The *kpsD* gene encodes a 60-kDa outer membrane protein that facilitates the transport of capsular polysaccharides across the outer membrane. The absence of *kpsD* causes the buildup of capsular polysaccharides in the periplasmic space and loss of surface expression of the capsule (35). As a control, CFT073 (K2 capsule) was also tested and shown to be resistant to K1-mediated phage lysis (Fig. 1A). We also performed a phage-killing assay, in which strains PA45B, PA45B*kpsD*, CFT073, and CFT073*kpsD* were cultured in the presence of K1 phage. Consistent with our analysis using the cross-brush method, the optical density at 600 nm (OD_600_) of wild-type PA45B was dramatically reduced when incubated in the presence of the K1 phage, whereas the unencapsulated PA45B*kpsD* mutant and CFT073 exhibited normal growth (Fig. 1B).

### Development of an assay to enrich for survival of capsule mutants.

To develop an assay to enrich for capsule mutants and identify capsule-associated genes by TraDIS, we tested different combinations of PA45B and mutant PA45B*kpsD* in mixed growth assays in the presence of K1 phage. We mixed approximately 2 × 10^8^ CFU of wild-type PA45B (~99%) with 2 × 10^6^ CFU of mutant strain PA45B*kpsD* (~1%) and incubated the culture with or without the K1 phage. Plating of the cultures on both normal LB plates and plates supplemented with chloramphenicol allowed us to determine the number of unencapsulated PA45B*kpsD* mutant colonies at different time points. In this assay, the percentage of PA45B*kpsD* mutant colonies steadily increased in the presence of K1 phage but remained essentially unchanged when no phage was added (Fig. S1). Taken together, these results demonstrate that K1-specific phage-mediated killing can be used to enrich for unencapsulated mutants in a mixed culture containing both encapsulated and unencapsulated cells.

10.1128/mBio.01558-17.1 FIG S1  Enrichment for unencapsulated PA45B mutants in a mixed culture following infection with K1 phage. Mixed cultures of wild-type PA45B and the mutant, complemented PA45B*kpsD* (*kpsD*::Cm) strain were incubated for 12 h with or without the K1 phage and then plated on both LB agar or LB agar supplemented with chloramphenicol. The percentage of chloramphenicol-resistant PA45B*kpsD* mutant colonies was determined by dividing the number of colonies on the LB-chloramphenicol plates (number of *kpsD*::Cm mutants) by the number of colonies found on the plain LB agar plates (total number of both wild-type PA45B and the PA45B*kpsD* mutant). Download FIG S1, TIF file, 0.9 MB. Copyright © 2017 Goh et al. 2017 Goh et al. This content is distributed under the terms of the Creative Commons Attribution 4.0 International license.

### Identification of genes associated with capsule production in PA45B.

**FIG 2:**
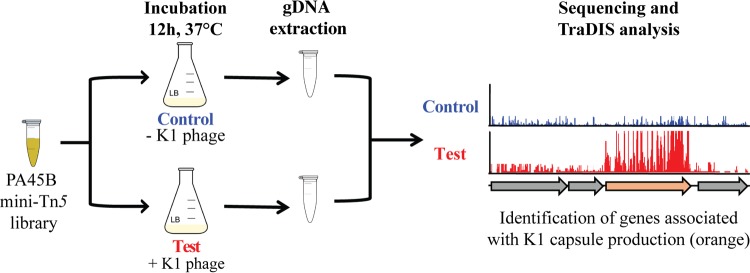
Experimental design to identify genes associated with K1 capsule production. Approximately 2 × 10^8^ PA45B mini-Tn*5* transposon mutants were added to 100 ml of LB broth with (test) or without (control) the K1 phage and incubated at 37°C for 12 h. PA45B genomic DNA was then purified and analyzed by TraDIS.

To facilitate a large-scale genetic screen for the identification of genes involved in K1 capsule synthesis by PA45B, we first generated a library of approximately 1 million mutants by using a mini-Tn*5* transposon carrying a chloramphenicol resistance gene. Next, we used this PA45B mutant library in our K1 phage-mediated killing assay to identify genes associated with capsule expression. In this assay, pools of roughly 2 × 10^8^ mutant bacteria were added to four different flasks containing 100 ml of LB broth, with two flasks containing the K1 phage (test pool) and two flasks with no phage (control pool). The phage treatment permitted the growth and enrichment of a small number of unencapsulated mutants, while the majority of bacteria that expressed the K1 capsule were lysed. After 12 h of incubation at 37°C, 1 ml of culture was extracted from each flask and washed once in phosphate-buffered saline (PBS) to remove any cell debris. PA45B genomic DNA was extracted from each pool and sequenced with a multiplex TraDIS strategy (Fig. 2). The test and control pools yielded a total of 6,042,698 Tn*5-*specific reads, of which 95.2% mapped to the PA45B genome. Further analysis of the control data revealed 430,791 unique insertion sites, which equates to one insertion approximately every 12 bp across the chromosome and emphasizes the high level of saturated mutagenesis and coverage in our library.

We screened for the enrichment of insertions in the test pool compared to the control pool, as mutants containing insertions in genes not related to capsule expression would be lost and thus underrepresented. Subsequently, a set of genes were identified to be involved in capsule production by our TraDIS analysis in which we used a stringent threshold cutoff of a log_2_ fold change (FC) in count reads of ≥5 and an adjusted *P* value of <0.001. This included the majority of genes within the K1 capsule gene cluster (*kpsFEDUCSMT* and *neuDBA*), two previously described regulators of capsule expression (*typA* and *rfaH*), a new regulator (mprA) (Table 1), and 12 other genes (Data Set S1E).

**TABLE 1:** Genes identified by TraDIS that affect capsule production in PA45B

**Gene name**	**Locus tag**	**Mutated gene**	**Capsule synthesis disrupted**^b^	**Genome positon**	**Product**	**Log FC**^c^
*mprA*	PA45B_3600	+	+	2953533–2954063	DNA-binding transcriptional repressor	7.28
*neuA^a^*	PA45B_3235	−	NA	3339754–3340863	*N*-Acylneuraminate cytidylyltransferase	7.21
*kpsC^a^*	PA45B_3240	−	NA	3336039–3337244	Capsule polysaccharide export protein	7.19
*kpsE^a^*	PA45B_3243	−	NA	3330382–3331530	Capsule polysaccharide export protein	7.18
*kpsD^a^*	PA45B_3242	+	+	3331554–333230	Capsule polysaccharide export protein	7.18
*kpsM^a^*	PA45B_3231	−	NA	3344548–3345324	Capsule polysaccharide transport protein	7.16
*kpsT^a^*	PA45B_3232	−	NA	3346382–3346918	Capsule polysaccharide transport ATP-binding protein	7.12
*kpsS^a^*	PA45B_3239	−	NA	3336039–3337244	Capsule polysaccharide export protein	7.10
*kpsF^a^*	PA45B_3244	−	NA	3329327–3330310	Capsule polysaccharide export protein	7.08
*neuB^a^*	PA45B_3234	−	NA	3342182–3343222	*N*,*N*′-diacetyllegion-aminic acid synthase	7.00
*neuD^a^*	PA45B_3233	−	NA	3343219–3343842	Galactoside *O*-acetyl-transferase monomer	7.00
*typA^a^*	PA45B_2253	+	+	4361535–4363358	GTP-binding protein	6.46
*kpsU^a^*	PA45B_3241	−	NA	3333240–3333980	Capsule polysaccharide export protein	5.38
*rfaH^a^*	PA45B_2283	+	+	4327933–4328421	DNA-binding transcriptional anti-terminator	5.08

^a^ Gene known to be involved in capsule expression.

^b^ NA, not applicable, because the gene was not mutated.

^c^ The log_2_ fold change in read counts from the test pool versus the control pool.

### Genetic characterization and validation of unencapsulated mutants.

**FIG 3:**
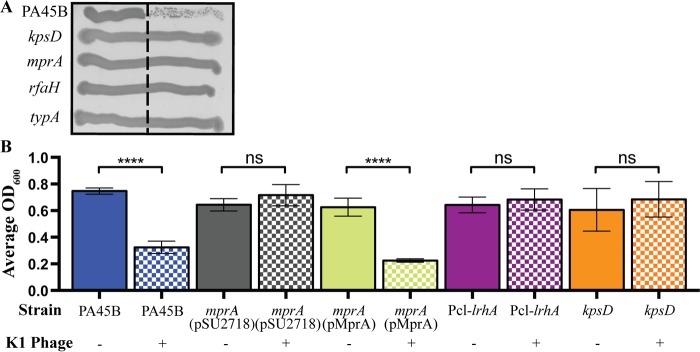
Expression of the K1 capsule in PA45B and isogenic mutants. (A) Cross-brush agar test results, showing K1 phage reactions of PA45B and isogenic *kpsD*, *mprA*, *rfaH*, and *typA* mutants. The dashed line indicates the line of phage suspension. (B) Measurement of susceptibility to phage lysis following growth in microtiter plates. The OD_600_ is shown 3 h postinfection. ****, *P* < 0.0001. Data were obtained from three independent experiments; error bars indicate standard deviations. Statistical analysis was performed using an unpaired, two-tailed *t* test.

To extend and confirm our TraDIS data, we generated a series of defined deletion mutants via λ Red-mediated recombination and tested the phage sensitivity of the mutants. The genes mutated included *rfaH*, *typA*, and *mprA* (Table 1), as well as 10 of the 12 other functionally uncharacterized genes identified by TraDIS (Data Set S1E). In this assay, only the PA45B *rfaH*, *typA*, and *mprA* mutants exhibited normal growth after crossing the phage suspension, indicating that these genes were required for capsule production (Fig. 3A; Fig. S2). The 10 other genes were regarded as false positives that were detected due to the strong positive selective pressure of our K1 phage-killing assay (see Discussion). We therefore focused our attention on the *mprA* gene and examined its role in capsule biosynthesis by cloning the gene into the expression plasmid pSU2718 (to construct plasmid pMprA) and transforming this plasmid into PA45B*mprA* to generate the complemented strain PA45B*mprA*(pMprA). The sensitivity to phage-mediated lysis was restored to the wild-type level in the PA45B*mprA*(pMprA) complemented mutant (Fig. 3B). Taken together, our data confirmed that MprA is required for expression of the K1 capsule in the UPEC strain PA45B.

10.1128/mBio.01558-17.2 FIG S2  Expression of the K1 capsule in PA45B and isogenic mutants. Cross-brush agar test results, depicting K1 phage interaction with PA45B and the corresponding isogenic mutants. The dashed line indicates the line of phage suspension. Download FIG S2, TIF file, 1.6 MB. Copyright © 2017 Goh et al. 2017 Goh et al. This content is distributed under the terms of the Creative Commons Attribution 4.0 International license.

### Insertions in intergenic regions associated with the loss of K1 capsule.

Our TraDIS data allowed us to identify the enrichment of insertions not only within genes but also within intergenic regions (IGR). We examined the impact of such insertions on the downstream genes, based on the orientation of the chloramphenicol resistance gene within the mini-Tn*5* transposon. We applied the same stringent threshold cutoffs as described above and identified eight IGRs that contained an increased amount of transposon insertions in the test pool compared to the control pool (Data Set S1F). Three IGRs were located immediately upstream of the capsule synthesis genes *kpsF*, *kpsE*, and *kpsM*. The unidirectional orientation of the transposon insertions in these loci (with the chloramphenicol resistance gene in the opposite direction to the downstream genes), coupled with the enrichment of insertions within these genes, suggested that transcription of the *kpsF*, *kpsE*, and *kpsM* genes was abolished. Indeed, these genes encode the transport and assembly machinery of the capsule, and hence they are essential for the surface expression of the capsular polysaccharide.

One IGR (IGR1287) contained a much higher number of unique mini-Tn*5* insertions than the other IGRs. In this IGR, all the mini-Tn*5* insertions were oriented in the same direction, with the chloramphenicol resistance gene placed in the same orientation as the downstream *lrhA* gene (Fig. S3). Moreover, no enrichment of insertions was observed within the coding sequence of *lrhA*. This suggested that there was enhanced transcription of *lrhA* via readthrough from the promoter of the chloramphenicol resistance gene (within the mini-Tn*5* transposon), which lead to increased levels of LrhA protein and ultimately loss of capsule expression. To confirm this hypothesis, we inserted a constitutive Pcl promoter upstream of *lrhA*, generating the strain PA45BPcl-*lrhA*. This strain was protected against K1 phage-mediated lysis (Fig. 3B), indicating that overexpression of *lrhA* abrogated the surface expression of the capsule. Taken together, our data show that LrhA is involved in K1 capsule regulation in PA45B.

10.1128/mBio.01558-17.3 FIG S3  Tn*5* insertion sites in PA45B chromosomal loci identified by TraDIS. Blue lines represent insertions identified from the control pool; purple lines represent insertions identified from the test pool. Lines in the top frame of each panel represent insertions with the promoter of the chloramphenicol resistance cassette pointing downstream, whereas lines in the bottom frame of each panel represent insertions with the promoter of the chloramphenicol resistance cassette pointing upstream. The heights of the lines indicate the number of insertions at a particular site. Red arrows represent genes which met the stringent threshold cutoff for the log_2_ FC of ≥5 count reads and an adjusted *P* value of <0.001. Indicated in each panel is the gene name as well as the log_2_ FC of the gene. (A) Insertions in genes that did not affect capsule expression. (B) Insertions in genes that affected capsule expression. Download FIG S3, PDF file, 0.8 MB. Copyright © 2017 Goh et al. 2017 Goh et al. This content is distributed under the terms of the Creative Commons Attribution 4.0 International license.

### Mutation of *mprA* and overexpression of LrhA decreases the transcription of the capsule genes.

**FIG 4:**
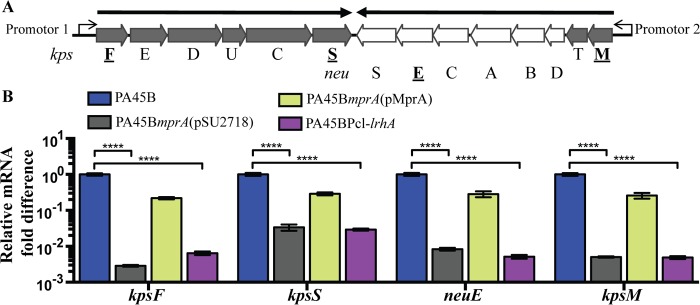
Expression of genes related to capsule synthesis. (A) Schematic diagram of the K1 capsule gene cluster. Dark gray, regions I (*kpsFEDUCS*) and III (*kpsMT*); white, region II (*neuDBACES*). The two bold arrows represent polycistronic transcripts initiating at promoters 1 (encompassing region I) and 2 (encompassing regions II and III). Underlined genes in bold represent genes examined via qRT-PCR. (B) qRT-PCR results, demonstrating the transcription levels of *kpsF*, *kpsS*, *neuE*, and *kpsM* in wild-type, mutant, and complemented strains. ****, *P* < 0.0001. Data were obtained from three independent experiments; error bars indicate standard deviations. Statistical analysis was performed using an unpaired, two-tailed *t* test.

To investigate the effects of MprA and LrhA on expression of the K1 capsule, we performed quantitative reverse transcriptase PCR (qRT-PCR) on the wild-type and mutant strains and examined the transcription of four genes within the capsule gene cluster: *kpsF*, *kpsS*, *kpsM*, and *neuE* (Fig. 4A). These genes represent the start and end of the polycistronic transcripts of the K1 capsule gene clusters. Mutation of *mprA* resulted in a significant decrease in the transcript levels of all four genes (between 17- and 200-fold decreases), whereas complementation with plasmid pMprA restored the transcription of these genes to wild-type levels (Fig. 4B). Similarly, the transcripts of all four genes were significantly decreased in the PA45BPcl-*lrhA* strain (between 20- and 179-fold decreases) (Fig. 4B). Taken together, these findings suggest that absence of MprA or overexpression of LrhA affects the transcription of genes within the capsule cluster.

### Mutation of *mprA* and overexpression of LrhA result in sensitivity to killing by factors in whole blood but do not affect the LPS.

**FIG 5:**
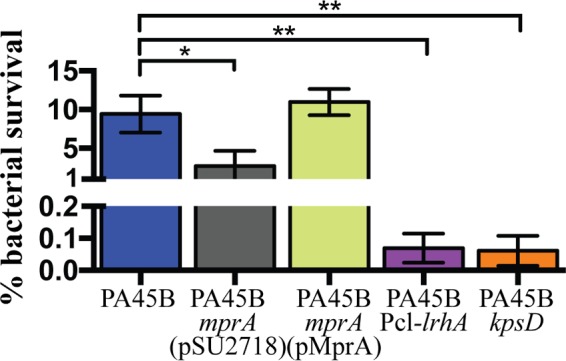
Survival of wild-type PA45B and isogenic mutants in whole-blood killing assays. Bacteria were incubated for 1 h in fresh whole blood, and colony counts were used to compare survival of mutants and complemented strains to that of the wild type. Survival of the *mprA*, Pcl-*lrhA*, and *kpsD* mutants was significantly reduced compared to wild-type PA45B. *, *P* = 0.0197; **, *P* = 0.0025. Data were obtained from three independent experiments; error bars indicate standard deviations. Statistical analysis was performed using an unpaired, two-tailed *t* test.

The capsule makes a key contribution toward UPEC virulence by conferring resistance against killing by innate host factors. Mutation of the capsule genes, or even enzymatic degradation of the K1 capsule, leads to increased susceptibility to complement-mediated killing (36). Hence, to verify altered expression of the capsule, we determined whether mutant strains were more susceptible to killing in whole blood. In these assays, the unencapsulated PA45B*mprA*, PA45BPcl-*lrhA*, and PA45B*kpsD* mutant strains displayed increased sensitivity to killing in whole blood compared to the PA45B wild-type strain (Fig. 5). Complementation of *mprA* in PA45B*mprA* restored survival to wild-type levels.

Bacterial LPS also contributes to the resistance of UPEC to human serum. To confirm the phenotype that we observed was due to the loss of K1 capsule synthesis and not altered LPS production, we extracted and analyzed the LPS profiles of the mutants by using Tricine-sodium dodecyl sulfate–polyacrylamide gel electrophoresis (TSDS-PAGE) and silver staining. In these experiments, a *waaL* mutant of PA45B was included as a control to depict an altered O antigen banding profile. The *waaL* gene encodes an O antigen ligase; mutation of this gene would prevent the attachment of the O antigen to the LPS core, and in our experiments this resulted in the loss of bands corresponding to O antigen subunits when cells were visualized by silver staining (Fig. S4). In contrast, we did not observe any difference in the O antigen banding profile between the mutant strains PA45B*mprA*, PA45B*mprA*(pMprA), and PA45BPcl-*lrhA* and wild-type PA45B (Fig. S4). Taken together, these results demonstrate that the increased sensitivities to killing in whole blood displayed by the unencapsulated *mprA* mutant and the LrhA-overexpressing strain are not due to modification of their LPS.

10.1128/mBio.01558-17.4 FIG S4  Analysis of LPS profiles via silver staining of PA45B wild-type and mutant strains. The lipid A core region is represented by the thick lower band. The bands stacked above the lipid A core represent the O antigen side chains. Download FIG S4, TIF file, 2.7 MB. Copyright © 2017 Goh et al. 2017 Goh et al. This content is distributed under the terms of the Creative Commons Attribution 4.0 International license.

### Mutation of *mprA* and overexpression of LrhA affect the motility of PA45B.

**FIG 6:**
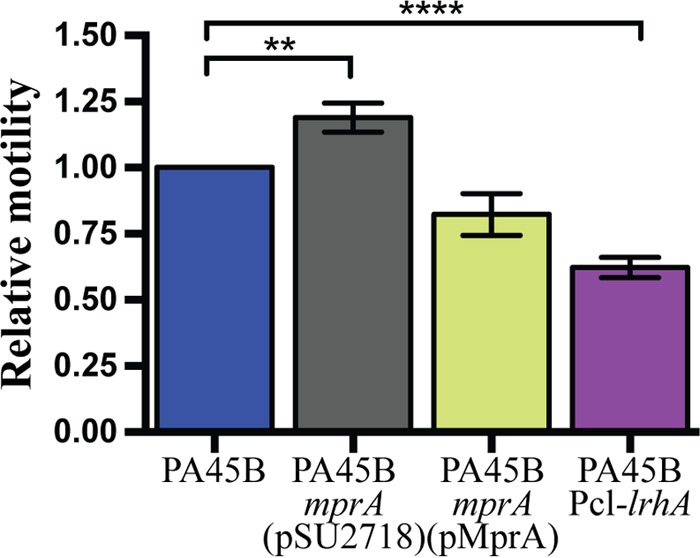
Motility phenotype of PA45B and isogenic mutants. The relative motility of each strain compared to that of wild-type PA45B was determined by measuring the diameter of the swimming zone after incubation at 37°C for 16 h. **, *P* = 0.0037; ****, *P* < 0.0001. Data were obtained from three independent experiments; error bars indicate standard deviations. Statistical analysis was performed using an unpaired, two-tailed *t* test.

Both MprA and LrhA have previously been shown to repress flagellum-mediated motility (37, 38). Therefore, we hypothesized that mutation of *mprA* would lead to increased motility, while overexpression of LrhA would reduce motility. To test this, we performed swimming assays and compared the motility of the mutant strains to that of wild-type PA45B. In these experiments, the PA45B*mprA* mutant displayed a small but significantly increased swimming phenotype compared to PA45B, and complementation with plasmid pMprA restored the swimming phenotype to wild-type levels. Conversely, strain PA45BPcl-*lrhA* displayed a lower level of motility than PA45B (Fig. 6). Collectively, our data suggest that while the loss of MprA or overexpression of LrhA in PA45B leads to the loss of K1 capsule production, these changes have opposite effects on motility; the loss of MprA results in an increased swimming phenotype of the strain, whereas overexpression of LrhA diminishes the swimming phenotype compared to wild-type PA45B.

### Mutation of *mprA* and overexpression of LrhA result in the loss of capsule expression in other UPEC strains.

**FIG 7:**
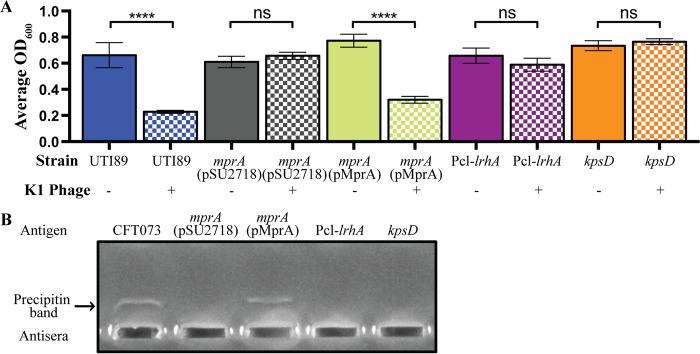
Expression of the capsule in UTI89 (K1) and CFT073 (K2). (A) Measurement of susceptibility to K1 capsule-dependent phage lysis following growth in microtiter plates. ****, *P* < 0.0001. Data were obtained from three independent experiments; error bars indicate standard deviations. Statistical analysis was performed using an unpaired, two-tailed *t* test. (B) Agarose gel showing the precipitin bands that occurred due to cross-linking of the K2 antigen and antisera postelectrophoresis. A positive reaction was observed with wild-type CFT073 and CFT073*mprA*(pMprA). No bands were observed for the *mprA*, Pcl-*lrhA*, and *kpsD* mutants.

To extend our findings on the roles of MprA and LrhA in capsule production, we first examined the role of these proteins in another UPEC strain possessing the same K1 capsule (strain UTI89). Similar to our observations for PA45B, mutation of *mprA* or overexpression of LrhA in UTI89 allowed the mutants to survive K1 phage-mediated lysis, whereas complementation of the UTI89*mprA* mutant strain with plasmid pMprA restored sensitivity to the K1 phage to wild-type levels (Fig. 7A). We next examined the role of *mprA* and *lrhA* in another UPEC strain expressing a different K capsule (strain CFT073 [K2]). Here, countercurrent immunoelectrophoresis with a K2-specific antiserum was used to detect the expression of the capsule. Precipitin bands were observed for capsular extracts prepared from wild-type CFT073 and strain CFT073*mprA*(pMprA) (i.e., the complemented mutant strain), whereas no band was detected from the CFT073*mprA* or CFT073Pcl-*lrhA* mutant strains (Fig. 7B).

**FIG 8:**
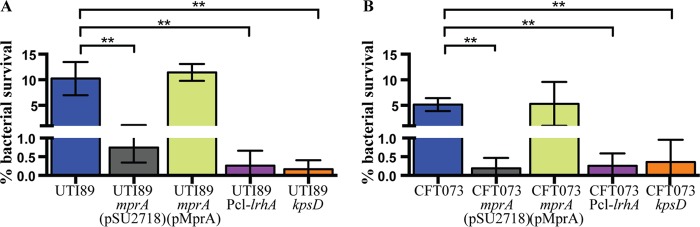
Survival of wild-type UTI89 and CFT073 and their corresponding isogenic mutants in whole-blood killing assays. Survival of the *mprA*, Pcl-*lrhA*, and *kpsD* mutants was significantly reduced compared to the wild-type strains, UTI89 (A) and CFT073 (B), respectively, after incubation in fresh whole blood. Survival of the *mprA* mutant was restored upon complementation with plasmid pMprA. **, *P* = 0.0029 to 0.0074. Data were obtained from three independent experiments; error bars indicate standard deviations. Statistical analysis was performed using an unpaired, two-tailed *t* test.

To investigate if the loss of capsule expression in UTI89 and CFT073 caused the same phenotype observed for PA45B, we subjected both strains, together with their respective mutants, to a series of whole-blood killing assays. Consistent with our previous observations, the unencapsulated mutants displayed increased sensitivity to killing in whole blood compared to their respective wild-type strains (Fig. 8). These results therefore demonstrated that the effect of mutating *mprA* or overexpressing LrhA on capsule production is not strain or capsule type specific.

## DISCUSSION

The role of the polysaccharide capsule is well established in UPEC virulence. The prototypical group 2 capsule provides protection against phagocytosis and complement-mediated killing (23, 24), contributes to immune evasion via molecular mimicry (33), and can also mask other surface-associated antigens (39). Group 2 capsule biosynthesis is controlled by a conserved export and assembly mechanism (16, 17, 19–22), suggesting that the expression of all group 2 capsules may be regulated by the same factors, hence providing an attractive target for the development of therapeutic agents.

We first examined a collection of 126 complete *E. coli* genomes to gain a better understanding of the distribution of the group 2 capsule types. We found that the majority of phylogroup B2 (75%; 33/44) and D (75%; 3/4) strains possess intact region I and III capsular export and assembly genes but carry an array of region II genes that encode different capsular K types. All 10 of the ST95 strains in our 126-strain database possess genes encoding a K1 capsule. ST95 is one of several major pandemic clonal lineages of UPEC found worldwide, and this sequence type has been noted for its relatively low frequency of drug resistance (4). Because our data suggested that the K1 capsule is closely associated with ST95 strains, it is possible that the K1 capsule may have provided a fitness advantage that contributed to the successful expansion of ST95. Moreover, the ST95 clonal group was first identified more than 70 years ago, suggesting that it is not a newly emerging clonal group undergoing drug resistance selection (4). Further work is now required to examine the distribution of K types in a larger collection of ST95 strains and to assess the impact of the K1 capsule on ST95 virulence.

Due to the poor immunogenicity of the K1 capsule and lack of readily available antisera, bacteriophages specific to the K1 capsule have been used as diagnostic tools to rapidly identify strains expressing the K1 antigen. These capsule-specific phages recognize and bind to the surface polysaccharide structure, allowing the phage to enter and subsequently kill the cell due to its lytic life cycle. Taking advantage of this phenotype, we exposed a highly saturated transposon mutant library to a commercially available lytic K1 capsule-specific phage, which allowed us to enrich for unencapsulated mutants in a mixed culture containing both encapsulated and unencapsulated cells. The use of a lytic phage in combination with TraDIS represents a novel approach to identify genes involved in capsule expression. Here, we searched for genes with a significant increase in insertion frequency in the output pool (test pool) compared to the input pool (control pool), as the disruption of these genes would have repressed capsule expression and hence conferred resistance to the K1 phage.

We initially hypothesized that all 14 genes within the capsule gene cluster (*kpsFEDUCSMT* and *neuDBACES*) would be identified in our screen. However, analysis of our TraDIS data revealed that three genes (*neuCES*) did not possess an enrichment of Tn*5* insertions and were not identified in our screen. The *neuC* gene encodes a UDP *N*-acetylglusoamine 2-epimerase which forms a sialic acid precursor and is required for the expression of the K1 capsule (40). The *neuS* gene encodes a polysialyltransferase which plays a role in assembling the full-length sialic acid polymer and is also required for K1 capsule expression (41). Although the exact role of NeuE has not been determined, NeuE, NeuS, and KpsC have been shown to be the minimum combination of proteins required to observe *de novo* polysialic acid synthesis in *E. coli in vitro* (41). We generated individual targeted deletion mutants of *neuC*, *neuE*, and *neuS* in PA45B and confirmed via a K1 phage assay that the mutants did not express the K1 capsule (Fig. S2). Closer examination of the three genes revealed that they all possessed a very low number of Tn*5* insertions, even in the control pool. Interestingly, all three genes have a lower GC content (*neuC*, 32.1%; *neuE*, 26.3%; *neuS*, 27.5%) than the entire PA45B genome (50.5%). The low GC content of these three genes could explain their absence from identification in the output pool, as the mini-Tn*5* transposon used to create the PA45B mutant library preferentially inserts into GC-rich regions (42). This has also been observed in another TraDIS study using the mini-Tn*5* transposon, where an overall increased transposon insertion frequency in high-GC versus low-GC regions was observed (43).

Besides the capsule gene cluster, our TraDIS analysis identified 15 other genes that could play a role in K1 capsule expression of PA45B. These included two genes encoding factors known to positively regulate capsule expression (*typA* and *rfaH*), which were confirmed in this study. The other 13 genes represented genes that may affect capsule expression, and we generated defined mutants of 11 of these genes for further investigation (despite multiple attempts, we were unable to generate defined *glpT_2* and *yghG* mutants in PA45B). Only the mutation of *mprA* in PA45B conferred resistance to the K1 phage that indicated the loss of capsule expression. Although this high false-discovery rate (FDR) seemed puzzling at first, further inspection of the Tn*5* insertions that mapped to these genes revealed that they possessed a much lower number of unique insertion sites than did other genes that were shown to affect capsule expression (Fig. S3). We therefore suggest that the discovery of these genes in our TraDIS experiment was due to independent random attenuation of capsule production (i.e., via spontaneous mutation or insertion of a second Tn*5* transposon into capsule biosynthesis or regulatory genes) combined with the extremely powerful positive selection invoked by the use of a lytic K1-specific phage. This highlights a previously unrecognized complication that should be considered when interpreting data associated with large-scale sequence-based genetic screening generated from experiments involving powerful positive selection. Further analyses confirmed the role of *mprA* in capsule expression: (i) transcription of the capsule genes was significantly decreased in the PA45B*mprA* mutant, (ii) the PA45B*mprA* mutant exhibited increased sensitivity to killing in whole blood, despite possessing an intact LPS, and (iii) complementation of the mutant strain PA45B*mprA* with the plasmid pMprA restored all phenotypes to wild-type levels.

MprA is a DNA-binding transcriptional regulator that belongs to the MarR family of winged-helix proteins. These proteins are involved in controlling the expression of multidrug efflux pumps and other virulence-associated factors in multiple pathogens (44). In *E. coli*, the *mprA* gene is located within an operon together with the *emrAB* genes, which encode a drug efflux pump that protects the bacterial cell from several antimicrobial agents (45). MprA represses transcription of the *emrAB* genes by directly binding to the promoter region of *emrA* (46). Mutation of *mprA* also leads to increased transcription of flagellar genes, increased expression of the FliC flagellin, enhanced flagellum synthesis, and a hypermotile phenotype (38). Recently, a study reported that small-molecule inhibitors of MprA prevented K1 polysaccharide capsule expression (47). Those authors showed that knockout of *mprA* had an effect similar to treatment with the inhibitor, with loss of encapsulation and complete attenuation in a murine sepsis model. They went on to show that although the *mprA* mutant exhibited an increase in *emrA* transcript levels, there was insufficient upregulation of the efflux pump to affect antibiotic resistance. However, those authors were unable to demonstrate direct binding of MprA to the capsule promoter regions, and bioinformatic analysis failed to identify any potential MprA-binding sites in these locations. Hence, it was concluded that the effect of MprA is likely indirect and possibly coordinated via a broader regulatory network. In this study, we used an unbiased forward genetic screen coupled with deep sequencing, and we also identified MprA as a factor affecting capsule expression. We showed that MprA is required for efficient transcription of the capsule genes and modulates the susceptibility to killing in whole blood. Our data confirmed the role of MprA in capsule expression, while the precise mechanism of how MprA affects capsule expression remains an area of ongoing investigation.

The highly saturated nature of the transposon mutant library used in our TraDIS analysis allowed us to identify not only transposon insertions within genes but also insertions in IGRs and the directionality of the insertions. The latter is important, as the promoter of the chloramphenicol resistance gene can drive the transcription of a downstream gene instead of disrupting it if the insertion position is favorable. There were eight IGRs that contained significantly more insertions in the output pool than in the input pool, which indicated that these insertions led to the loss of capsule expression. Four IGRs contained transposon insertions with the promoter of the chloramphenicol resistance gene oriented away from the downstream genes, suggesting that these insertions disrupted transcription of the respective genes. Three of these IGRs with insertions oriented away from the downstream genes were located upstream of genes known to be required for capsule production, and they were identified in our TraDIS screen. Another one such IGR was found immediately upstream of *mprA*, providing further evidence for the role of MprA in capsule production. In contrast, another IGR contained a significant increase in transposon insertions oriented toward the downstream *lrhA* gene. The lack of insertions within *lrhA* suggested that the transposon insertions likely caused increased transcription of the gene. We confirmed this increased transcription by inserting a constitutive Pcl promoter upstream of *lrhA* to drive strong transcription of the gene, and we found that the PA45BPcl-*lrhA* strain was also resistant to the K1 phage. Mutation of *lrhA* did not impact K1 capsule expression (Fig. S2). However, the PA45BPcl-*lrhA* strain exhibited a decrease in the transcription of capsule genes and an increased sensitivity to killing in whole blood. We noted that both the PA45BPcl-*lrhA* and PA45B*kpsD* strains were exquisitely sensitive to killing in whole blood, while strain PA45B*mprA*, although also sensitive, survived in slightly higher numbers. The reason for the difference in sensitivity to killing in whole blood remains unknown but may reflect the modes of action of LrhA and MprA. LrhA is a DNA-binding transcriptional regulator that belongs to the LysR family of proteins (48). It regulates the transcription of genes involved in motility, chemotaxis, and flagellum synthesis by repressing the expression of the master regulator FlhDC, and it also represses the expression of the sigma factor RpoS, which in turn prevents the transcription of hundreds of stationary-phase genes (37). Additionally, LrhA represses the expression of type 1 fimbriae, an adhesin important for colonization of the bladder and a key factor contributing to biofilm formation on abiotic surfaces and IBC formation within superficial cells of the bladder urothelium (49, 50). Overall, our data demonstrate that LrhA represses capsule production, and we speculate that this occurs at the transcriptional level via direct binding to the promoter regions of the capsule biosynthesis genes, or by enhancing binding of the RNA polymerase holoenzyme, mechanisms that have been previously attributed to LrhA function (51, 52).

We showed that both MprA and LrhA affect capsule expression and motility, albeit in different ways, and may contribute to different phases of bacterial lifestyles (Fig. S5). The absence of MprA results in cells that are unencapsulated and hypermotile. The capsule can also block the function of short adhesins through physical shielding (39). We speculate that a subpopulation of hypermotile, unencapsulated bacteria could aid in dispersal/ascension within the urinary tract, where fimbrial and afimbrial adhesins could contribute to adherence and colonization at new sites. However, overexpression of LrhA results in unencapsulated cells that are less motile than the wild type. LrhA also regulates a number of factors involved in biofilm formation, including type 1 fimbriae, flagella, and the stationary-phase sigma factor RpoS (50, 53). This suggests that varied intracellular levels of LrhA may lead to the development of subpopulations of cells primed to adapt to different steps in biofilm formation (i.e., adherence, microcolony development, maturation) and dispersal (54), as well as resistance to host innate immune factors and colonization. MprA and LrhA likely contribute to this lifestyle adaptation, as they regulate multiple factors involved in UPEC virulence.

10.1128/mBio.01558-17.5 FIG S5  Effect of MprA and LrhA on bacterial lifestyles. (A) Schematic diagram depicting the possible impact of MprA and LrhA regulators on several major UPEC virulence factors. Thick black lines, flagella; thins lines with rounded ends, T1F; dark gray oval, capsule; red lines, autotransporter (AT) proteins. The capsule can mask surface-associated AT proteins, such as Ag43. (B) When external stimuli cause the repression of MprA, expression of flagella is upregulated while capsule expression is repressed. Enhanced motility provides UPEC with the ability to swim toward other cells or surfaces. Loss of capsule expression may unmask AT proteins that can mediate cell-to-cell aggregation and biofilm formation. Expression of flagella can also aid in the dispersal of cells. (C) Overexpression of LrhA represses the capsule, flagella, type 1 fimbriae (T1F), and the stationary-phase sigma factor RpoS. This results in unencapsulated UPEC, which may form cell aggregates or biofilms through the action of AT proteins, such as Ag43, or other cryptic fimbriae. As the concentration of LrhA within the cell decreases, UPEC may shift toward increased expression of factors that contribute to epithelial cell invasion and IBC formation (i.e., the RpoS sigma factor, capsule, T1F). In the dispersal mode, flagellated UPEC may detach and disseminate to new sites. Download FIG S5, TIF file, 1.5 MB. Copyright © 2017 Goh et al. 2017 Goh et al. This content is distributed under the terms of the Creative Commons Attribution 4.0 International license.

Group 2 capsules share common regulatory elements; the *mprA* and *lrhA* genes identified in this study are highly conserved and are found within the *E. coli* core genome (55). This suggests that the effects of MprA and LrhA may be similar across all strains expressing a group 2 capsule. Indeed, we demonstrated that mutation of *mprA* and overexpression of LrhA in two other UPEC strains (UTI89 and CFT073) also caused the loss of capsule expression. Thus, the molecular control of capsule synthesis mediated by these two regulators is neither strain nor capsule K type specific.

Overall, our study highlights the combined power of saturated transposon mutagenesis and deep sequencing as a highly efficient forward genetic screening method to study bacterial virulence. Despite the fact that group 2 capsules have been studied for decades, our combined use of a lytic K1 capsule-specific phage and TraDIS uncovered new aspects of K1 capsule regulation and expression. A better understanding of the precise molecular mechanisms that govern how the genes identified in this work regulate capsule expression is now needed.

## MATERIALS AND METHODS

### Ethics approval.

Approval for the collection of human blood for whole-blood killing assays was obtained from the University of Queensland Medical Research Ethics Committee (2008001123).

### Bioinformatic analysis.

The *E. coli* database was represented by 126 published complete genomes available on the NCBI database as of May 2016. Kaptive was used to assess the prevalence of group 2 capsules (56). Sequence comparisons were examined using the FASTA36 software package (57). The *E. coli* strains were classified into major phylogroups (A, B1, B2, D, E, and F) based on an *in silico* analysis of the *arpA*, *chuA*, *yjaA*, and TSPE4.C2 loci (58). Multilocus sequence typing analysis was performed using the sequences of seven housekeeping genes as previously described (59).

### Bacterial strains and culture conditions.

Cells were routinely cultured shaking at 37°C in Luria-Bertani (LB) broth medium that was supplemented, when appropriate, with the following antibiotics: gentamicin (Gent; 20 μg/ml), ampicillin (Amp; 100 μg/ml), kanamycin (Kan; 50 μg/ml), or chloramphenicol (Cm; 30 μg/ml). Expression of genes was induced with either 1 mM isopropyl β-d-1-thiogalactopyranoside (IPTG) or 0.2% l-arabinose when required. All strains and plasmids used are outlined in Data Set S1G.

### Molecular methods.

Methods for DNA extraction, purification, sequencing, and PCR were previously described (60). Deletion mutants were constructed using the λ-Red recombinase gene replacement system as described previously (61); primers are listed in Data Set S1H. For qRT-PCR, exponentially growing cells (500 µl; OD_600_ of 0.6) were stabilized in 1 ml of RNAprotect bacteria reagent (Qiagen). Subsequent RNA extraction, DNase I treatment, first-strand cDNA synthesis, and qRT-PCR were performed as previously described (60). Gene expression levels were determined with the cycle threshold (2^−ΔΔ*CT*^) method (62), with differences expressed relative to the wild-type PA45B response. All experiments were performed in three independent replicates.

### Genome sequencing and assembly.

PA45B was sequenced on a Pacific Biosciences (PacBio) 249 RSII system using the P4 polymerase and C2 sequencing chemistry. The raw sequencing data were assembled as previously described (43). Annotation of the PA45B genome sequence was performed using PROKKA v1.11 (63) and the EcoCyc database (64), and the sequence was visualized in Artemis (65). The K1 capsule gene cluster from UTI89 was used to manually annotate the capsule gene cluster in PA45B.

### K1 phage assay.

A K1 polysialic acid capsule-dependent lytic phage (5.6 × 10^11^ PFU/ml) sourced from the Statens Serum Institut (Denmark) was used. To determine phage titers, 50 µl of PA45B culture (OD_600_ of 1.0) was first added to 3 ml of warm soft agar, spread onto LB agar plates, and allowed to dry. Serial dilutions of the phage suspensions (5 µl) were spotted onto the plates, and the numbers of plaques were determined following overnight incubation at 37°C. The K1 phage suspension was used to identify encapsulated cells in two assays. First, the cross-brush method was performed as recommended by the manufacturer (11). Inhibition of growth after crossing the phage suspension line was considered a positive reaction. For the second assay, overnight cultures of wild-type or mutant PA45B cells were subcultured 1:100 into fresh LB broth in microtiter plates and incubated with shaking at 37°C. After 90 min, the plates were removed from the shaker and 2 µl of the K1 phage suspension was added to each well. The plates were returned to the shaker for another 3 h, and the OD_600_ was measured to determine the extent of phage-mediated lysis.

### Generation and screening of the PA45B mini-Tn*5* library.

Generation of the PA45B mini-Tn*5* library was performed as previously described (66). The final library of approximately 1 million mutants was generated by pooling three batches of mutants, each containing approximately 160,000 to 580,000 mutants. Approximately 2 × 10^8^ bacterial cells from the PA45B mini-Tn*5* library were inoculated into 100 ml of LB broth either with (test) or without (control) 3 µl of K1 phage. After 12 h of shaking incubation at 37°C, 1 ml of culture was removed, washed with PBS once, and resuspended in 1 ml of PBS. Genomic DNA was extracted from this cell suspension using the Ultraclean microbial DNA isolation kit (Mo Bio Laboratories). The screening assays were performed in duplicate.

### Multiplex TraDIS.

Genomic DNA from each sample (tests and controls) was subjected to library preparation by using the Nextera DNA library prep kit (Illumina) with slight modification to amplify and sequence Tn*5* insertion sites (43). Briefly, 50 ng of genomic DNA was fragmented and enzymatically tagged with an adapter sequence (“tagmentation”) and then purified using the Zymo DNA clean and concentrator kit (Zymo Research). The PCR enrichment step, for which we used a custom transposon-specific primer to enrich for transposon insertion sites and an index primer (one index per sample) to allow for multiplexing sequencing, was performed at 72°C for 3 min and 98°C for 30 s, followed by 22 cycles of 98°C for 10 s, 63°C for 30 s, and 72°C for 1 min. Each library was purified using Agencourt Ampure XP magnetic beads. Library verification and quantification were undertaken using a Qubit 2.0 fluorometer and 4200 Tapestation system (Agilent Technologies). All libraries were pooled and submitted for sequencing on the MiSeq platform at the Australian Centre for Ecogenomics (University of Queensland, Australia).

### Analysis of TraDIS data.

Raw sequencing reads from TraDIS analysis were filtered and trimmed to keep 30 bp immediately after the 12-bp Tn*5*-specific tag (5′-TATAAGAGACAG-3′) at their 5′ ends by using the FASTX toolkit (http://hannonlab.cshl.edu/fastx_toolkit/index.html). These reads were aligned to the PA45B genome (CP021288) using Bowtie (version 1.1.2) (67) with its default arguments, and aligned reads were reported with “-M 1 --best” parameters. Subsequent analysis steps were carried out in R (version 3.3.1), with the Rsamtools package (version 1.26.1) (68) to calculate the number of sequence reads (read counts) and the number of insertion sites per gene (and intergenic regions), which were then used to estimate the log FC and FDR by use of the edgeR_package (version 3.16.1) (69, 70). To identify genes required for K1 capsule expression, we used stringent criteria of log FC of ≥5, FDR of ≤0.001, and read count of any site within a gene not exceeding 1/3 of total reads mapped to that gene.

### Whole blood killing assay.

Approximately 10^7^ CFU in 100 µl, prepared from a late exponential-phase culture, was added in duplicate to 900 µl of whole blood collected from a healthy donor by using Vacuette tubes containing sodium heparin (Grenier Bio-one). The samples were incubated with rolling at 37°C for 1 h and then plated in triplicate onto LB agar. The survival rate was expressed as the percentage of surviving cells compared to the initial inoculum.

### Biochemical and phenotypic assays.

*E. coli* K2 antiserum (Statens Serum Institut) was used to detect the presence of the K2 antigen, and capsule extracts were made as previously described (26). Countercurrent immunoelectrophoresis was performed for 70 min at 80 V in 1× Tris-acetate-EDTA buffer, and a precipitin band between the wells indicated the presence of the K2 capsule. LPS was extracted and resolved using Tricine-SDS–PAGE as described previously (66, 71). For motility assays, 6 µl of an overnight culture was spotted onto freshly prepared 0.25% LB Bacto agar plates in triplicate and incubated at 37°C for 16 h in a closed box containing a beaker of water to prevent drying. The rate of motility was expressed relative to motility of wild-type PA45B (i.e., diameter of the motility zone of mutants divided by the diameter of the motility zone of wild-type PA45B).

### Accession number(s).

The sequences for the PA45B chromosome and pPA45B plasmid have been deposited in the NCBI GenBank database under accession numbers CP021288 and CP021289, respectively. The raw PacBio sequence reads have been deposited in the Sequence Read Archive (SRA) under accession number SRR5585696. The TraDIS reads have been deposited at the SRA under accession numbers SRR5520531, SRR5520532, SRR5520533, and SRR5520534.
