# Supplementary figures and images for: Genome-Wide Discovery of Genes Required for Capsule Production by Uropathogenic Escherichia coli
Source: mBio. 2017 Oct 24;8(5):e01558-17. doi: 10.1128/mBio.01558-17 (PMC5654933; doi:10.1128/mBio.01558-17)

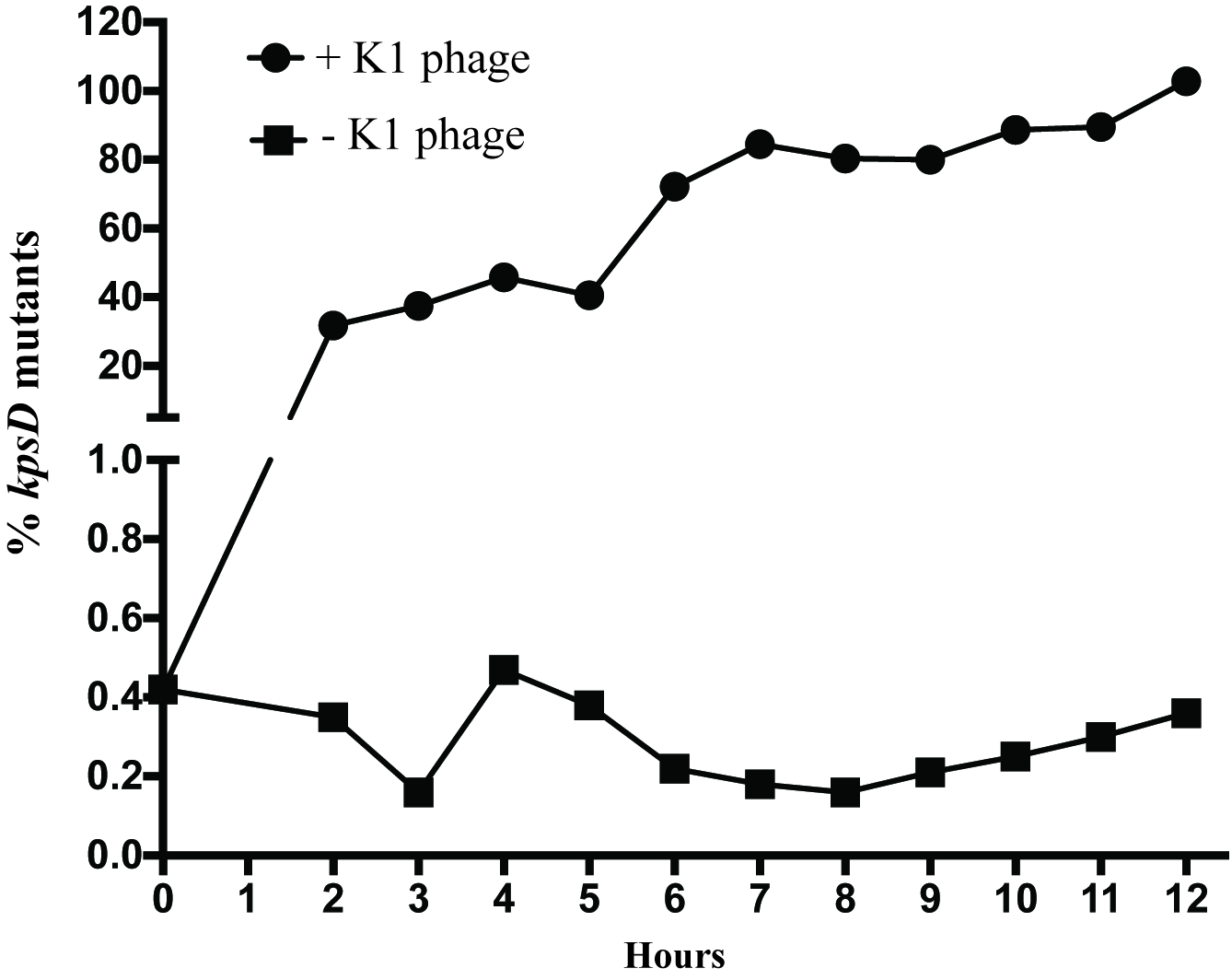

Supplement: FIG S1 [file mbo005173539sf1.tif]

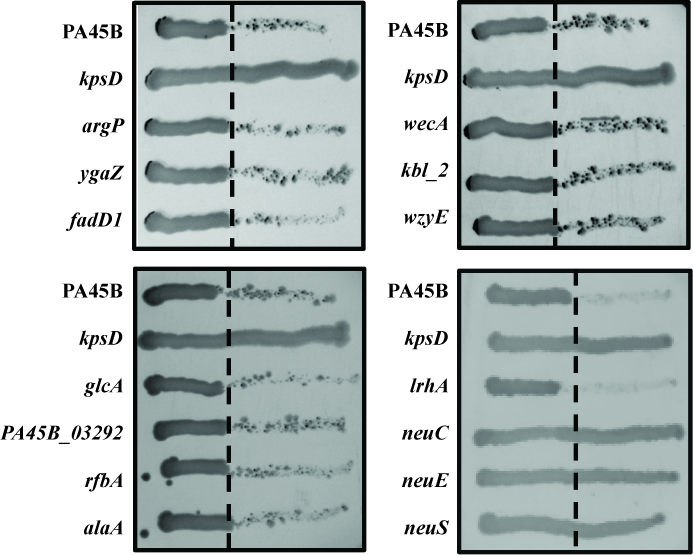

Supplement: FIG S2 [file mbo005173539sf2.tif]

A

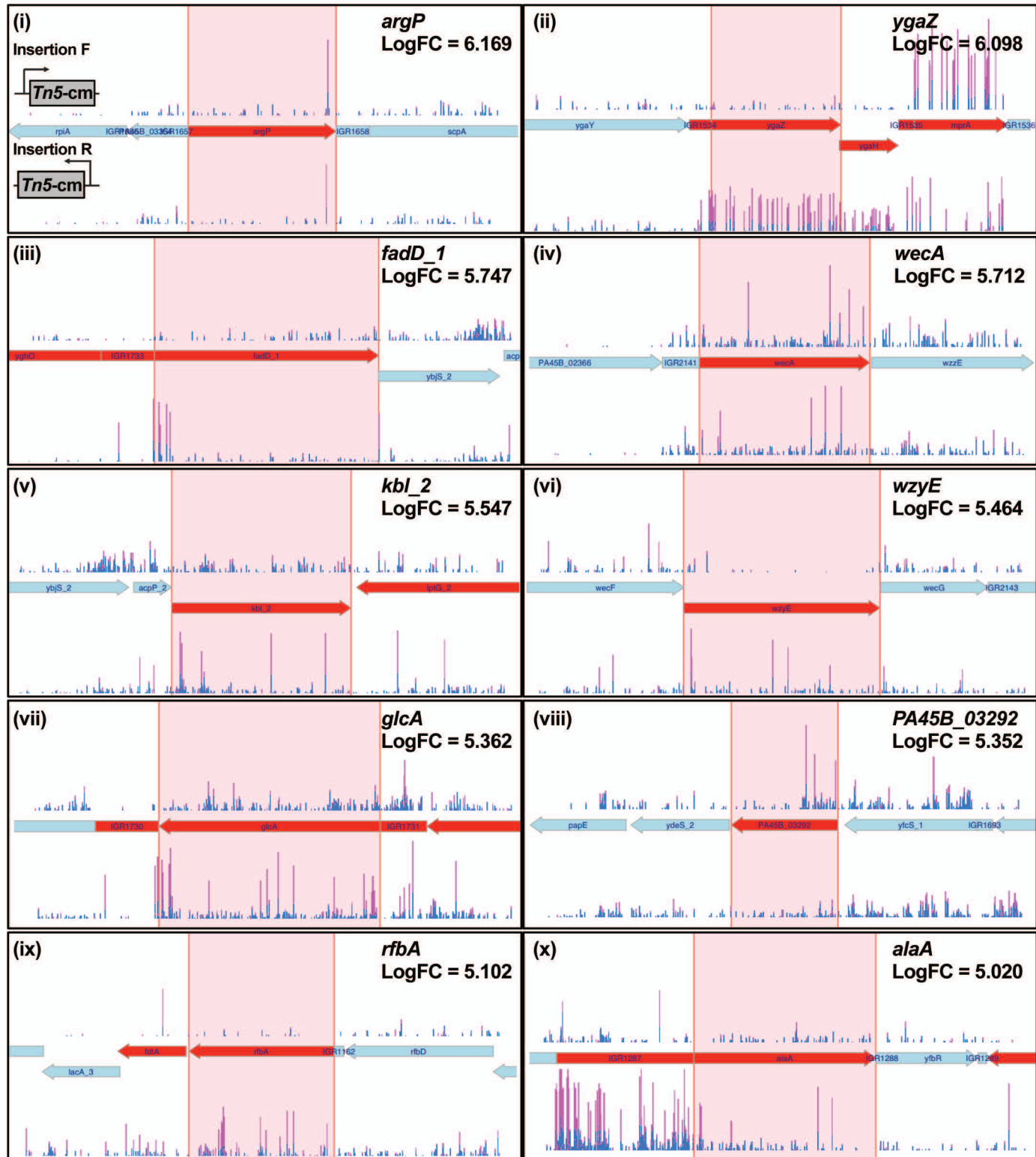

B

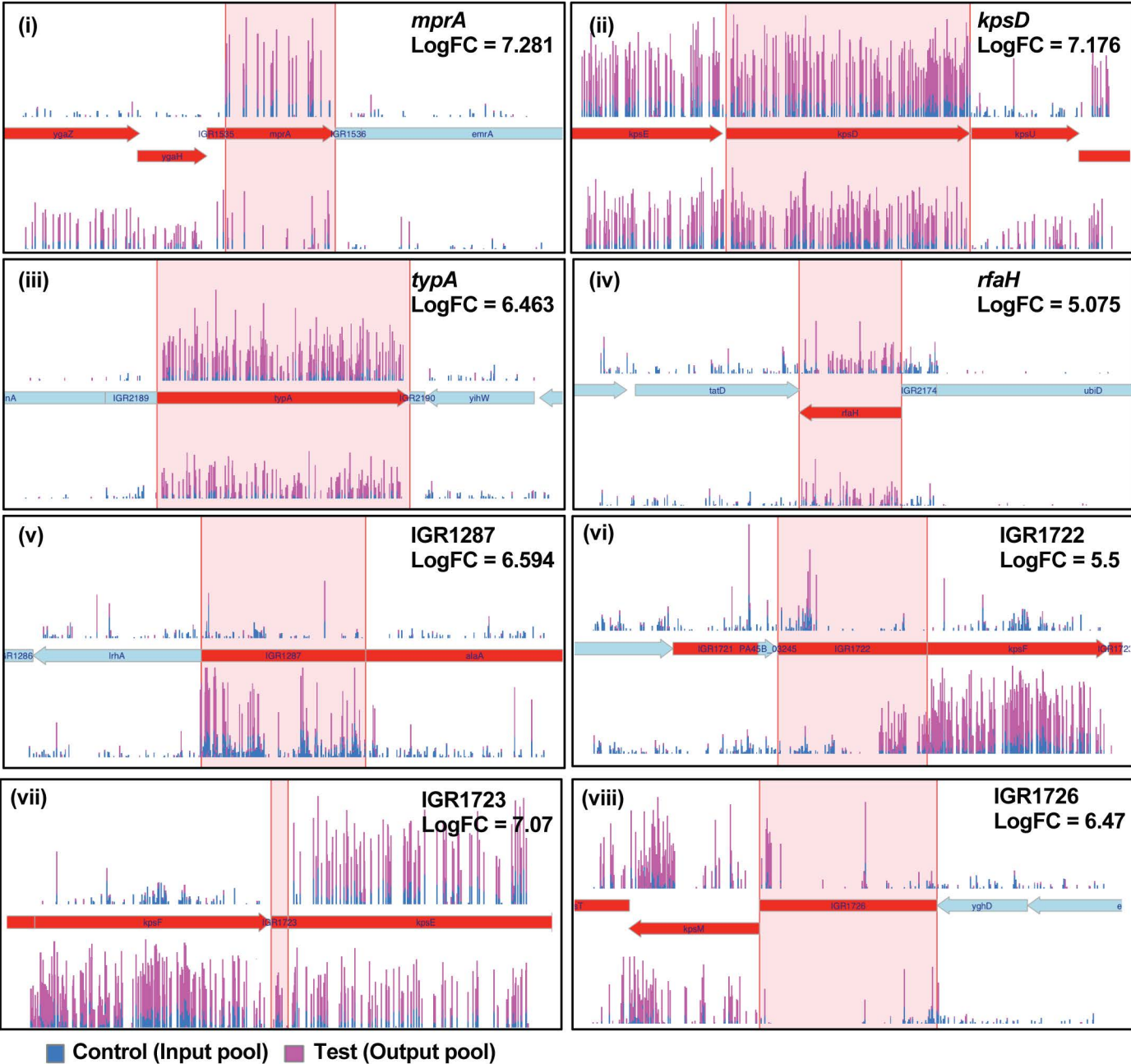

Supplement: FIG S3 [file mbo005173539sf3.pdf]

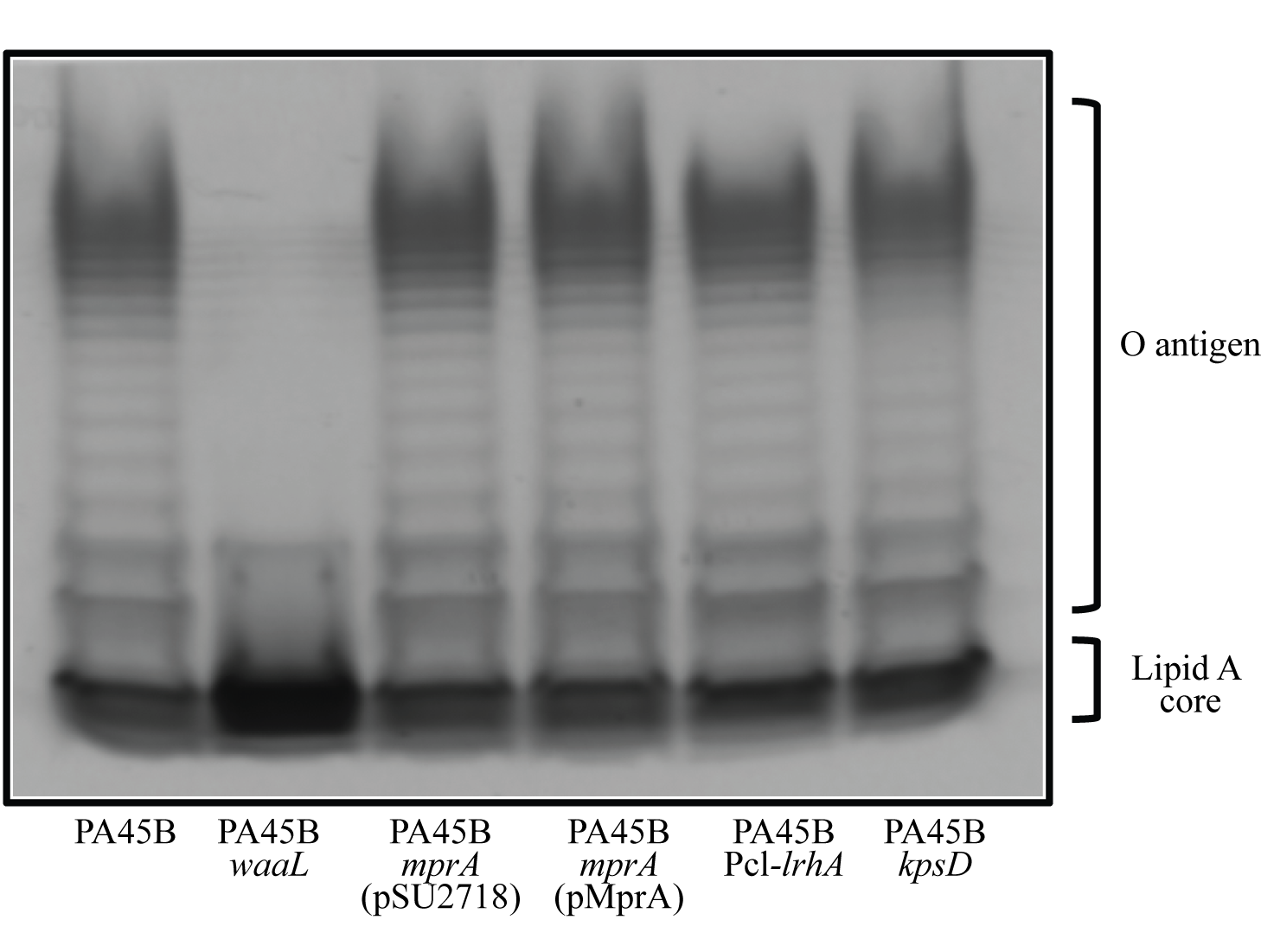

Supplement: FIG S4 [file mbo005173539sf4.tif]

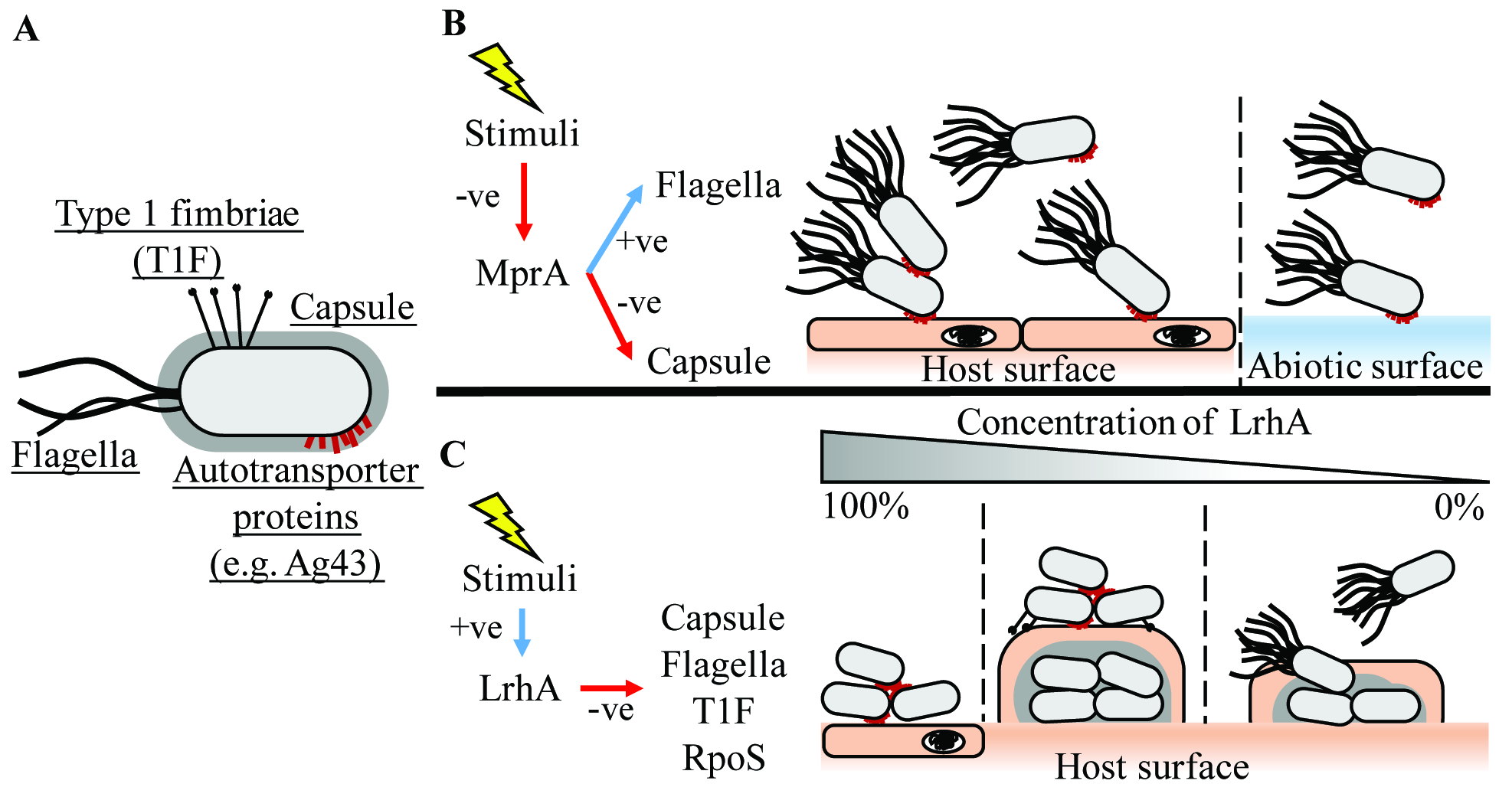

Supplement: FIG S5 [file mbo005173539sf5.tif]
